# Multi Vessel Coronary Artery Disease Presenting as a False Negative Myocardial Perfusion Imaging and True Positive Exercise Tolerance Test: A Case of Balanced Ischemia

**DOI:** 10.7759/cureus.11321

**Published:** 2020-11-04

**Authors:** Abdul Baqi, Intisar Ahmed, Babar Nagher

**Affiliations:** 1 Cardiology, Aga Khan University Hospital, Karachi, PAK; 2 Cardiology, Aga Khan University Hospital, Karachi, PAK

**Keywords:** balanced ischemia, myocardial perfusion imaging, exercise tolerance test

## Abstract

Non-invasive investigations play an important role in the early diagnosis of coronary artery disease. Although the stress test is an easily available investigation for the diagnosis of obstructive coronary artery disease, its results are affected by the pretest probability. Myocardial perfusion imaging (MPI) is one of the commonly performed non-invasive cardiac imaging. The common reasons for false-negative exercise MPI are reported to be inadequate exercise, a lower dose of radio-tracer, small area of perfusion defect, and ischemia caused by left circumflex or its branches. Balanced ischemia is one of the rare causes of false-negative MPI. In this case report, we present a 73-year-old gentleman who presented with chest pain and shortness of breath. An exercise electrocardiogram (ECG) at five metabolic equivalents was positive for symptoms and electrocardiographic evidence of ischemia, but the myocardial perfusion image did not show ischemia. An invasive coronary angiogram was done due to high-risk exercise ECG, which revealed severe three-vessel coronary artery disease.

Although the false-negative myocardial perfusion scan in the presence of a positive exercise electrocardiogram is unusual, it should not be ignored. Positive exercise ECG with the reproduction of symptoms indicates a high probability of critical coronary artery disease, irrespective of perfusion defects.

## Introduction

Coronary artery disease is the leading cause of mortality and morbidity in developed countries and its prevalence is growing exponentially in developing as well as under-developed countries [1]. Non-invasive investigations play an important role in the early diagnosis of coronary artery disease. Although stress test is the easily available and most commonly used investigation for the diagnosis of obstructive coronary artery disease, its results are affected by the pretest probability of the disease [2]. In patients with a low pretest probability of coronary artery disease, it has high rates of false-positive, and in those with high pretest probability, there are higher chances of false negatives. That is why it is recommended in patients with an intermediate pretest probability of coronary artery disease [3]. Myocardial perfusion imaging (MPI) is one of the commonly performed non-invasive cardiac imaging which has an additional role of detecting myocardial viability on top of establishing the diagnosis and prognosis of patients with coronary artery disease [4]. The radio-tracer used in myocardial perfusion imaging is distributed in the myocardium and a gamma camera detects the photons emitted by the radiotracer. The uptake of radio-tracer is dependent on perfusion, poorly perfused myocardium shows low uptake and well-perfused myocardium reveals high uptake of the radio-tracer [5,6]. Although myocardial perfusion imaging has high sensitivity, a false-negative test is not uncommon. The common reasons for false-negative exercise MPI are reported to be inadequate exercise, a lower dose of radio-tracer, small area of perfusion defect, and ischemia caused by left circumflex or its branches [7]. Here we report a case of a patient having a false negative myocardial perfusion scan in the setting of multi-vessel coronary artery disease on invasive coronary angiogram.

## Case presentation

A 73-year-old gentleman presented to the cardiology clinic with chest pain on moderate exertion for the last two weeks, which was relieved at rest. He also complained of shortness of breath on moderate exertion with no history of resting symptoms and was asymptomatic at the time of presentation. He was recently diagnosed to have chronic lymphocytic leukemia (CLL) and had not been started on any treatment yet. He did not have any risk factors for coronary artery disease except advanced age and male gender.

**Figure 1:**
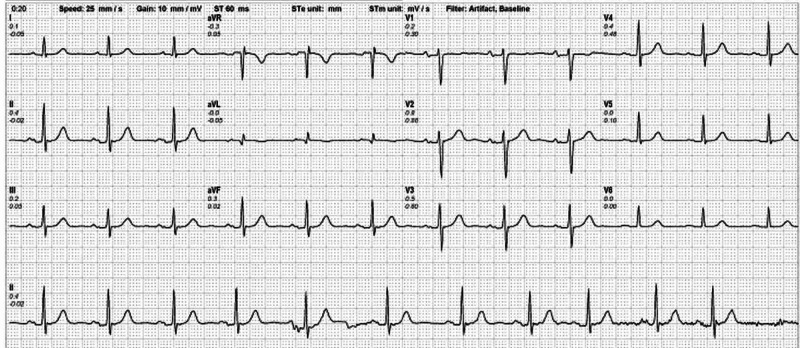
Resting electrocardiogram (ECG) shows normal sinus rhythm with no changes suggestive of ischemia

On examination, he had a heart rate of 84 beats per minute, blood pressure of 135/75 mmHg, respiratory rate of 16 breaths per minute and an oxygen saturation of 98% while breathing in room air. Resting electrocardiogram (ECG) revealed sinus rhythm with no significant ischemic changes (Figure 1). 

Other investigations revealed hemoglobin of 13.9 g/dl, total leukocyte count of 70.7x10E9/L, platelets count of 177 x10E9/L, serum creatinine of 0.7 mg/dl, serum sodium of 143 mmol/L, and hemoglobin A1c (HbA1c) was 5.8 %. His total serum cholesterol level was 159 mg/dl, high-density lipoprotein (HDL) was 28 mg/dl and low-density lipoprotein (LDL) level was 92 mg/dl. Trans-thoracic echocardiogram (TTE) showed an ejection fraction (EF) of 55% with no segmental wall motion abnormality and there was no structural abnormality.

**Figure 2:**
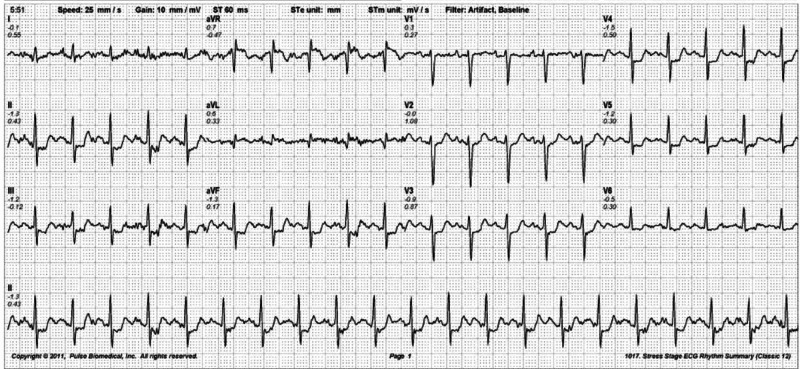
Electrocardiogram (ECG) at peak exercise showing more than 1 mm horizontal ST depression in precordial and inferior leads along with ST elevation in lead aVR.

He was advised to get a stress myocardial perfusion imaging (MPI) for the assessment of ischemia. He underwent single-photon emission cardiac tomography (SPECT) with Technetium 99-m (Tc-99). The patient exercised for four minutes on the Bruce protocol and achieved 84% of the maximum predicted heart rate. Technetium-99 was injected at 84% of the age-predicted maximum heart rate. The test was stopped due to exercise limiting chest pain and dyspnea. ECG at peak stress showed >1mm horizontal ST depressions in leads II, III, aVF, and V3 to V5 along with ST elevation in lead aVR (Figure 2). ECG changes and symptoms were relieved at six minutes of recovery.

**Figure 3:**
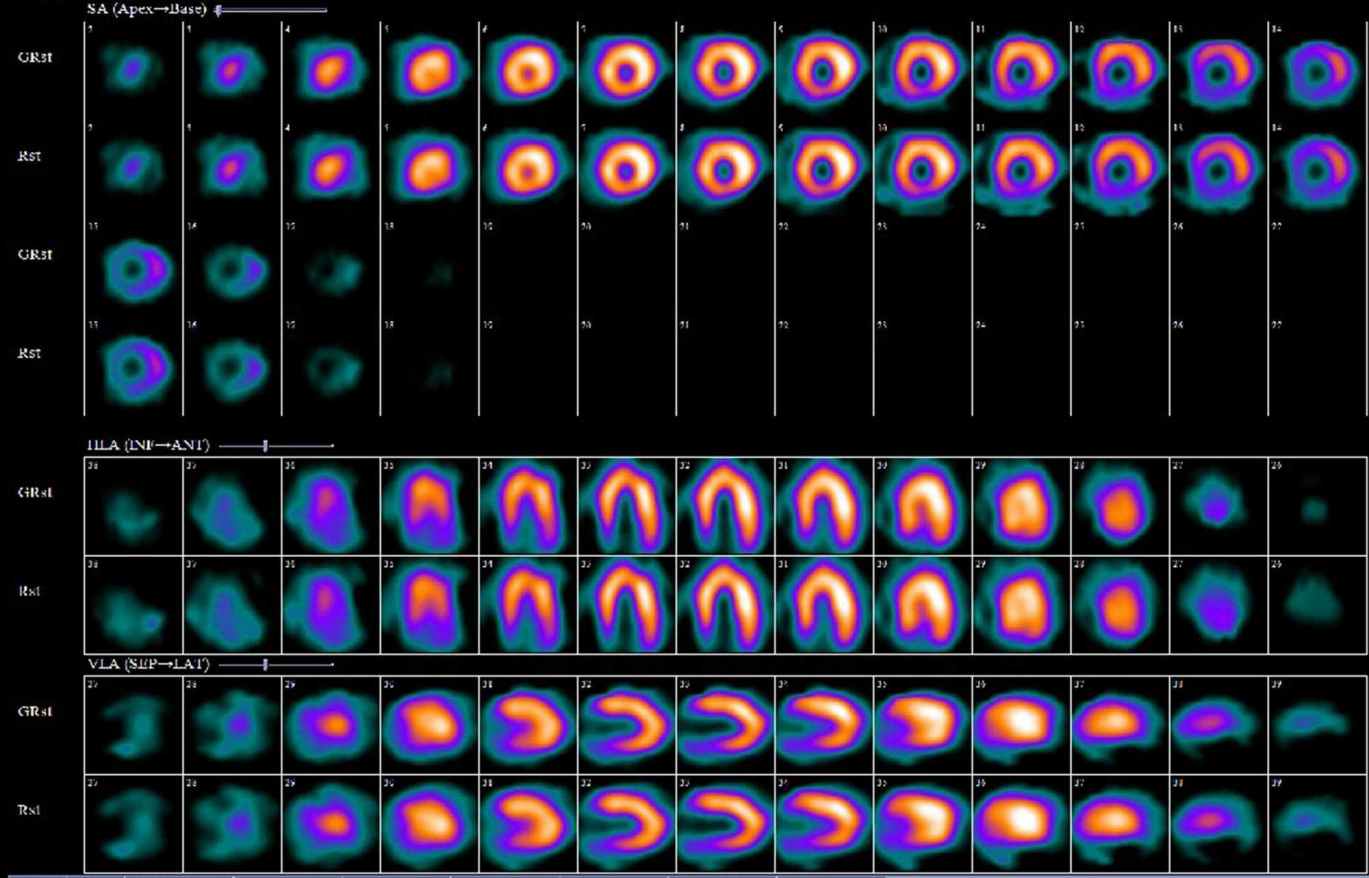
Myocardial perfusion scan showing small sized fixed perfusion defect involving the basal inferior segment.

Myocardial perfusion imaging (MPI) revealed a small-sized fixed perfusion defect of moderate-intensity in the basal inferior segment (Figure 3).

**Figure 4:**
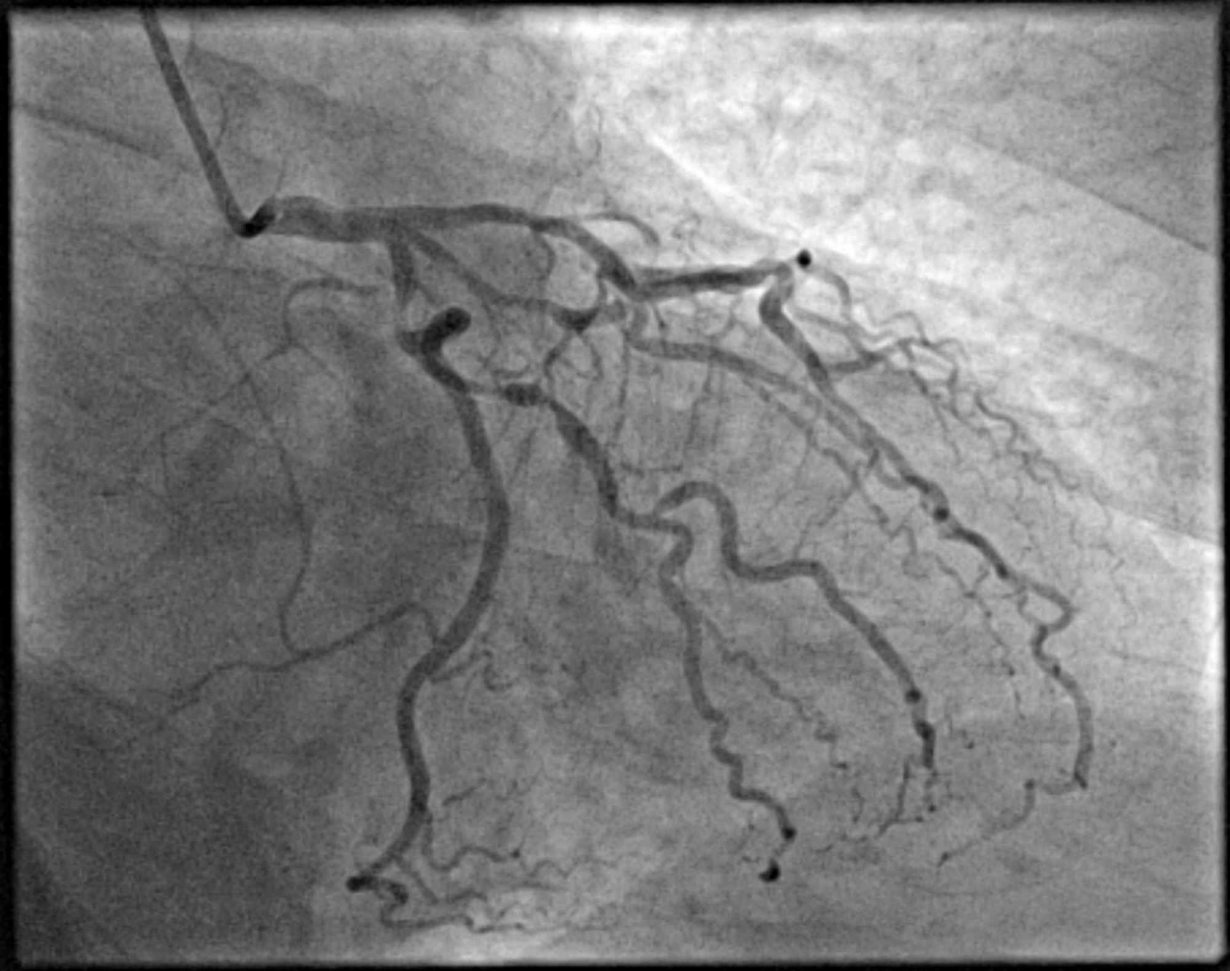
Caudal projection showing an obstructive lesion in the proximal segment of LCx, OM1, and OM2 branches. LCx (Left circumflex), OM1 (Obtuse marginal 1), OM2 (Obtuse marginal 2)

**Figure 5:**
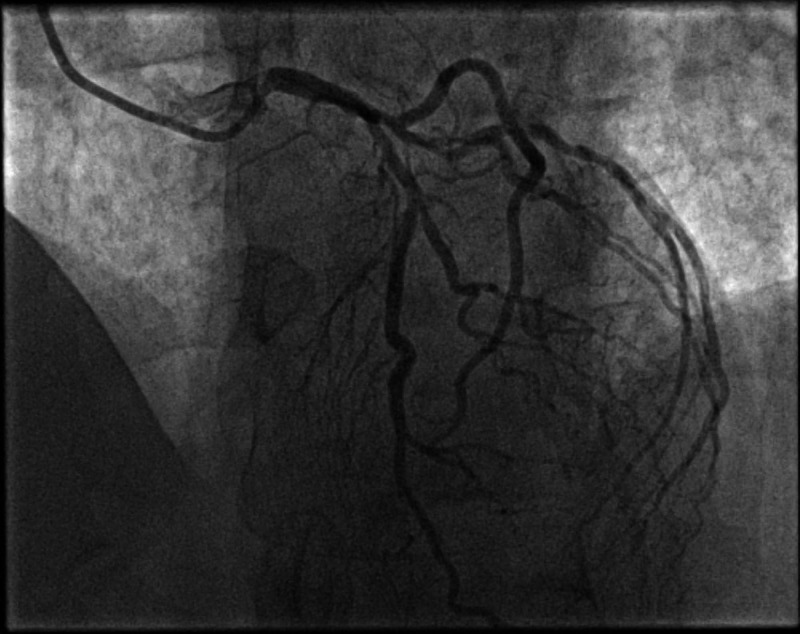
Cranial projection showing a significant obstructive lesion in the proximal and mid segments of the left anterior descending artery.

**Figure 6:**
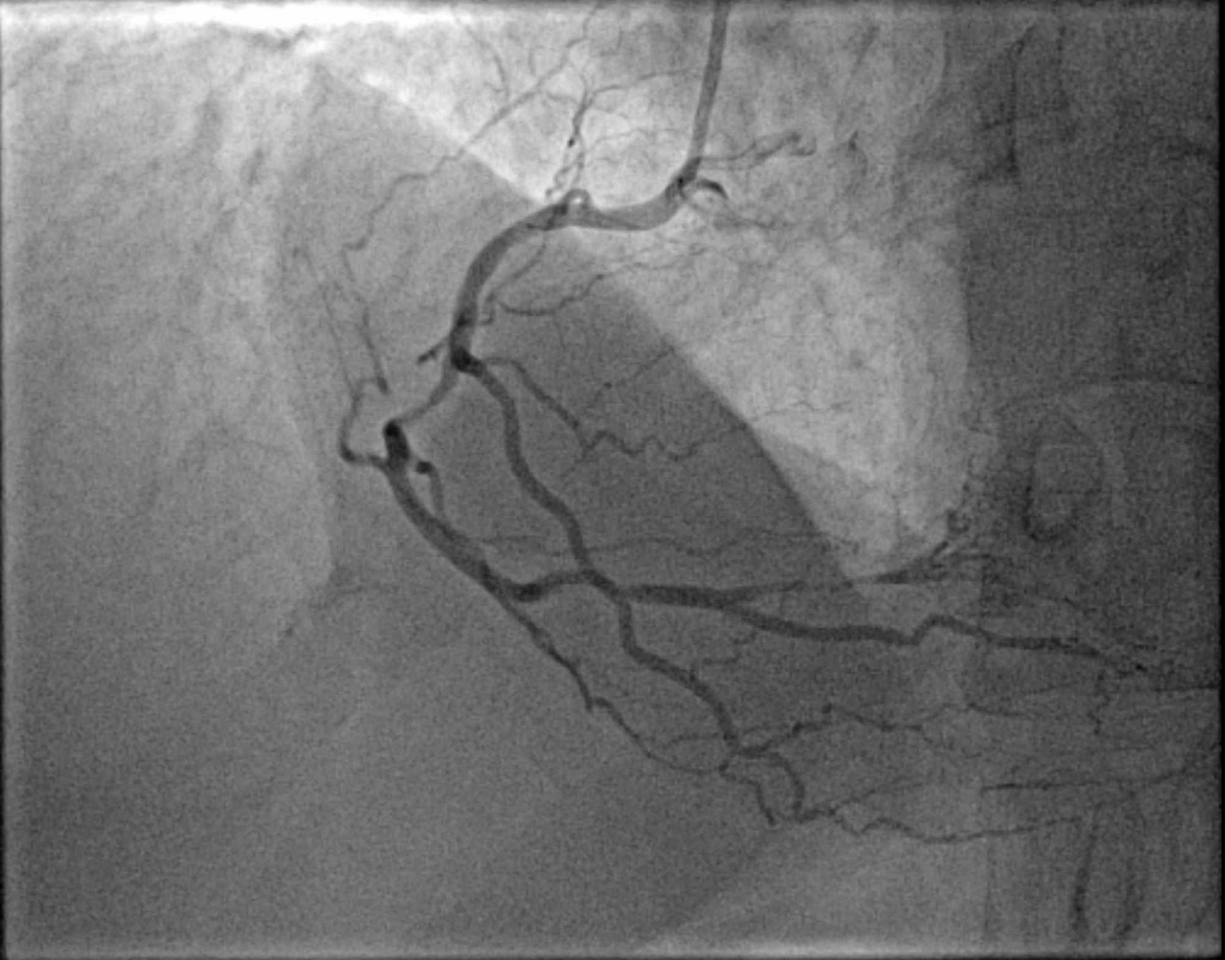
Cranial projection of the right coronary artery, showing obstructive lesions in the mid-segment of RCA and RPLV branch. RPLV (right posterolateral ventricular), RCA (right coronary artery)

Although MPI did not show any ischemia, exercise ECG was positive for symptoms and electrocardiographic evidence of myocardial ischemia. He was started on aspirin, rosuvastatin, bisoprolol, and then underwent an invasive coronary angiogram, which revealed multi-vessel coronary artery disease (Figures 4-6).

His current treatment was continued and long-acting isosorbide mononitrate added to his treatment regimen. After a detailed discussion with the patient and family members, he was referred to a cardiac surgeon for coronary artery bypass graft.

## Discussion

In this case report, we described a patient with chest pain and shortness of breath, who was found to have electrocardiographic evidence of ischemia on exercise ECG but MPI did not show ischemia. Invasive coronary angiogram confirmed the diagnosis of severe multi-vessel coronary artery disease.

The exercise stress test is a major noninvasive diagnostic test for coronary artery disease, which is readily available and easy to interpret. A combination of exercise ECG and myocardial perfusion imaging has significantly increased the diagnostic accuracy of stress myocardial perfusion imaging [8].

Although SPECT MPI is reported as one of the most sensitive non-invasive tests for coronary artery disease and patients with negative MPI have been studied to have less than 1% incidence of major cardiovascular events for a year [9]. However, normal MPI has also been reported in patients with angiographically significant coronary artery disease. The possible causes of false-negative MPI in that situation may include branch vessel stenosis, left circumflex artery stenosis, inadequate exercise, and caffeine intake before MPI (for dipyridamole myocardial perfusion imaging) [10,11,12].

Balanced three-vessel coronary artery disease has been reported as one of the rare causes of false-negative myocardial perfusion scans [13]. Our patient had severe three vessels coronary artery disease and false-negative MPI. But exercise electrocardiogram was positive for ischemia and his symptoms were reproduced on even low level of exercise, which makes a duke treadmill score of -11.5, suggesting a high likelihood of coronary artery disease and high risk of future cardiovascular events [14].

The detection of ischemia on MPI is based on relative perfusion of different myocardial segments. The segment with maximum uptake is considered normal and other segments are compared with that. In the presence of balanced ischemia, although radiotracer uptake is reduced overall. It is equally distributed in this setting and is proposed to appear due to the unopposed apical ischemia thus considered diagnostic [15].

## Conclusions

Although the false-negative myocardial perfusion scan in the presence of a positive exercise electrocardiogram is unusual, it should not be ignored. Positive exercise ECG with the reproduction of symptoms indicates a high probability of critical coronary artery disease, irrespective of perfusion defects.
